# Supplementary material for: In utero pulse injection of isotopic amino acids quantifies protein turnover rates during murine fetal development
Source: bioRxiv. 2023 May 21:2023.05.18.541242. Preprint. [Version 1] doi: 10.1101/2023.05.18.541242 (PMC10245746; doi:10.1101/2023.05.18.541242)
Supplement: Supplement 7 [file NIHPP2023.05.18.541242v1-supplement-7.pdf]

bioRxiv preprint doi: https://doi.org/10.1101/2023.05.18.541242; this version posted May 21, 2023. The copyright holder for this preprint (which was not certified by peer review) is the author/funder, who has granted bioRxiv a license to display the preprint in perpetuity. It is made available under aCC-BY 4.0 International license.

Supplementary Figures:

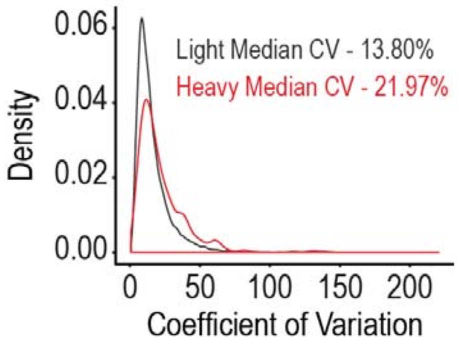

Figure S1: Peptide variability

Coefficient of variation (CV) for light and heavy peptides using six technical replicates. The coefficient of variation was calculated using normalized peptide abundances in the linear scale.

bioRxiv preprint doi: https://doi.org/10.1101/2023.05.18.541242; this version posted May 21, 2023. The copyright holder for this preprint (which was not certified by peer review) is the author/funder, who has granted bioRxiv a license to display the preprint in perpetuity. It is made available under aCC-BY 4.0 International license.

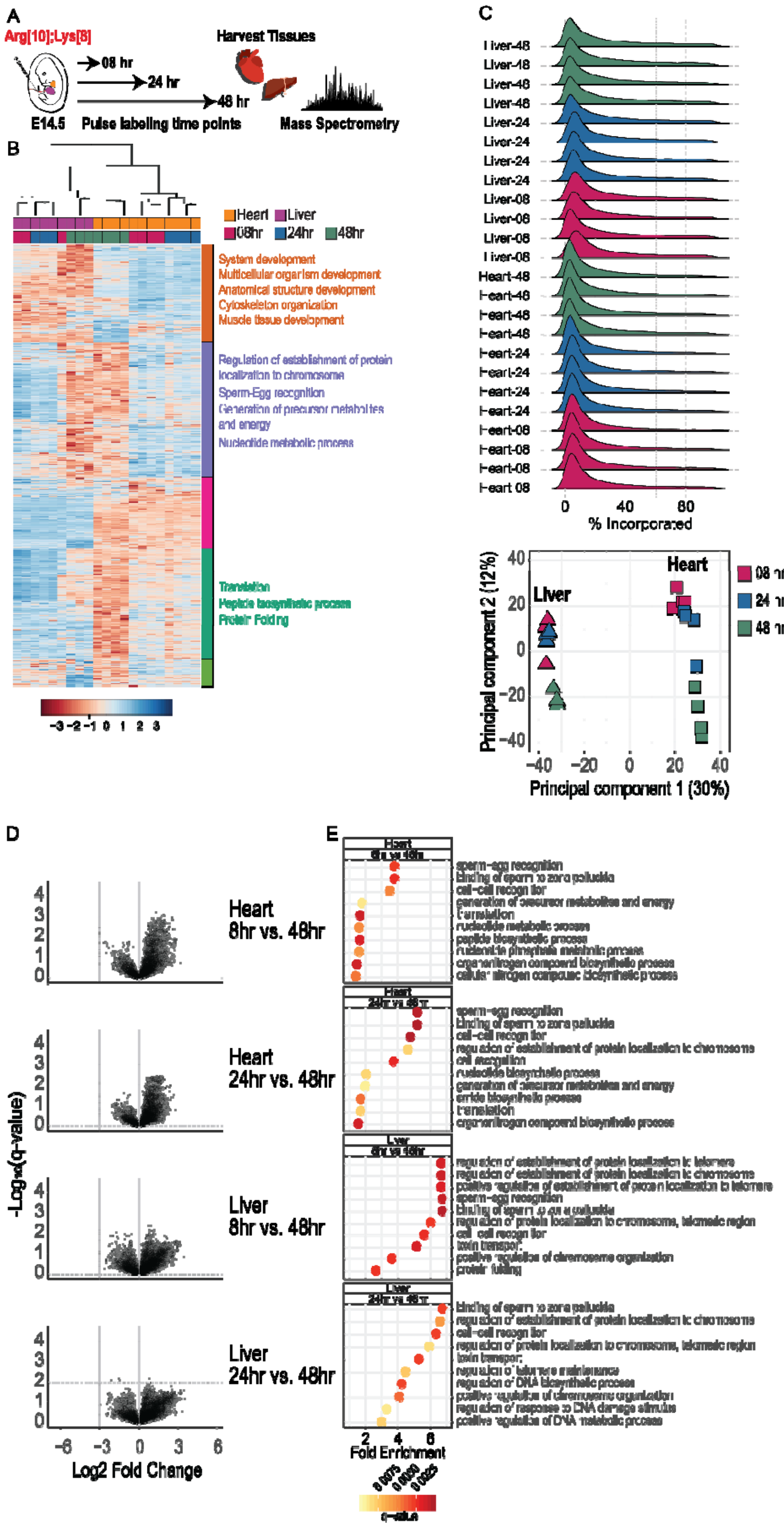

bioRxiv preprint doi: https://doi.org/10.1101/2023.05.18.541242; this version posted May 21, 2023. The copyright holder for this preprint (which was not certified by peer review) is the author/funder, who has granted bioRxiv a license to display the preprint in perpetuity. It is made available under aCC-BY 4.0 International license.

Figure S2: Labeling kinetics of labeled amino acids.

A. Experimental design for assessing labeled amino acid incorporation. E14.5 fetal mice are pulse injected with labeled arginine (Arg10) and lysine (Lys8) followed by a labeling period of 8, 24, and 48 hours. Heart and liver tissues are harvested and analyzed by mass spectrometry. **B.** Heatmap of the heavy peptide abundances for all labeling periods in heart and lung tissues. **C.** Distribution of heavy amino acid incorporation for all labeling periods in the heart and liver. **D.** Volcano plot comparing the heavy peptide abundance between heart and liver. Statistical analysis was performed using a Student's t-test and p.values were adjusted for multiple hypothesis testing. **E.** Gene ontology of the significant hits for each comparison.

bioRxiv preprint doi: https://doi.org/10.1101/2023.05.18.541242; this version posted May 21, 2023. The copyright holder for this preprint (which was not certified by peer review) is the author/funder, who has granted bioRxiv a license to display the preprint in perpetuity. It is made available under aCC-BY 4.0 International license.

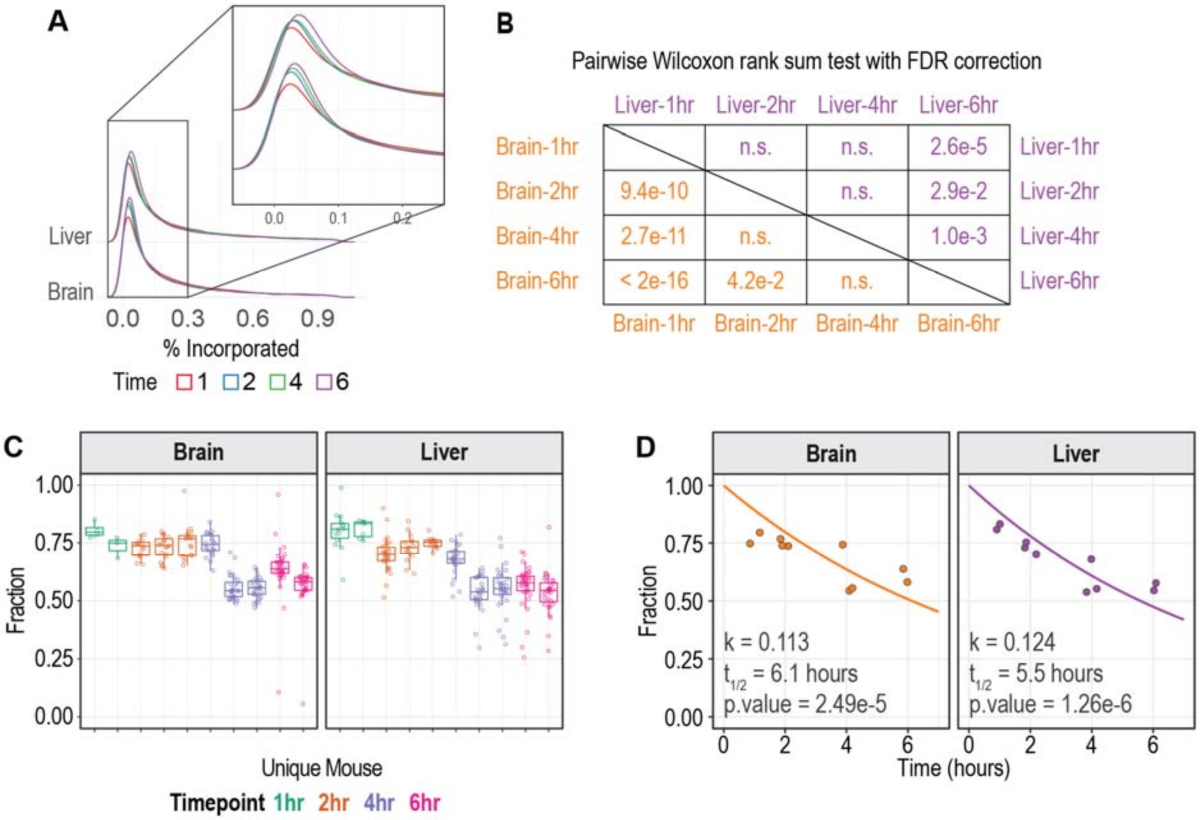

Figure S3: Validation of in utero turnover quantification

A. Distribution of heavy labeled amino acid incorporation into the fetal tissue proteome across the different time points. **B.** Pairwise comparison of the incorporation distribution from A. Wilcoxon rank sum test with FDR correction was used for the pairwise comparison. **C.** Relative isotope abundance (RIA) of the precursor amino acid pool quantified by mass isotopomer distribution analysis (MIDA). Peptides containing two lysines or two arginines for each sample were assessed to quantify the RIA as described previously[24,30]. Individual points in the boxplots represent the calculated RIA value for unique peptides across samples and time points. **D.** The median RIA value from each sample in C. was used for a pulse-chase kinetic model.

bioRxiv preprint doi: https://doi.org/10.1101/2023.05.18.541242; this version posted May 21, 2023. The copyright holder for this preprint (which was not certified by peer review) is the author/funder, who has granted bioRxiv a license to display the preprint in perpetuity. It is made available under aCC-BY 4.0 International license.

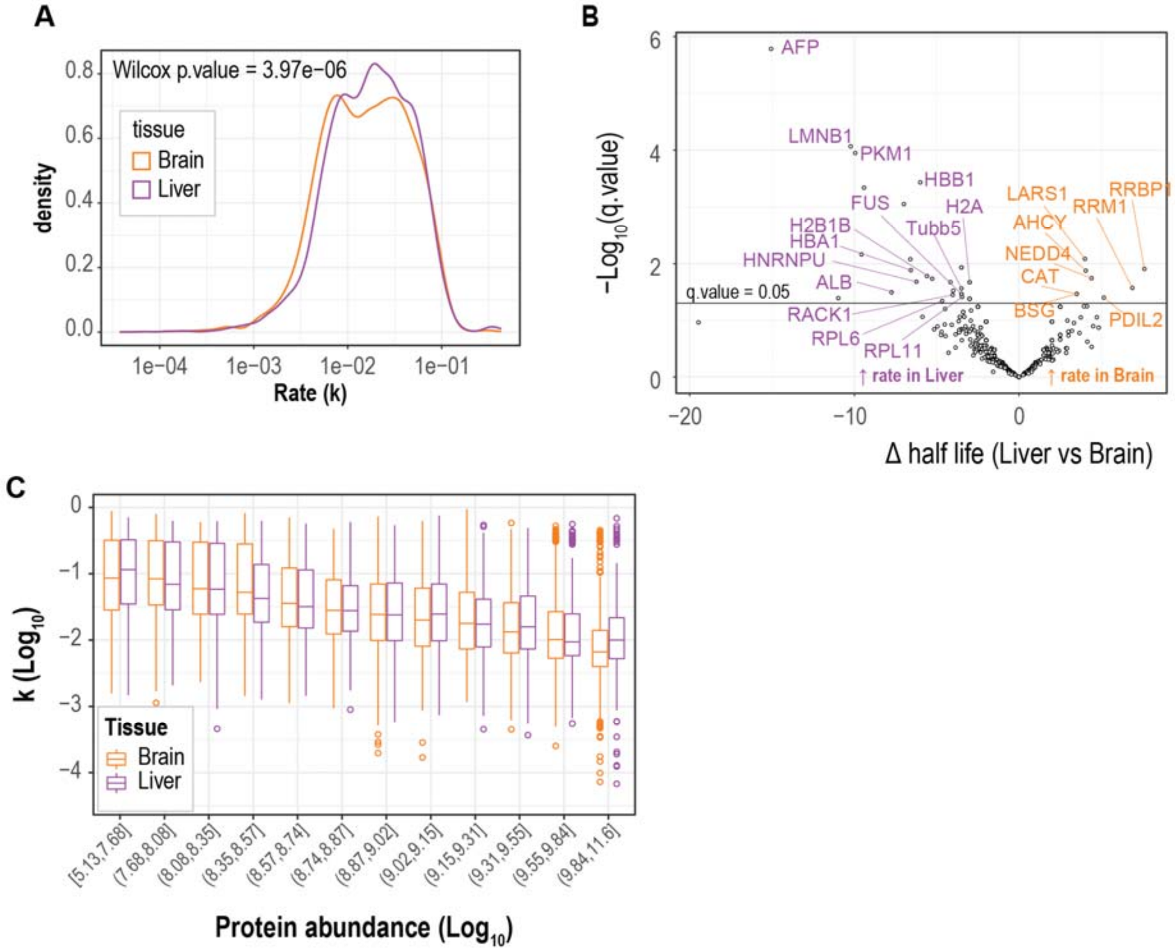

Figure S4: Liver and brain tissue turnover analysis.

A. Distribution of quantified turnover rate in liver and brain tissues. A Wilcoxon rank sum test was used to compare the distributions. B. Volcano plot comparing half-lives of proteins between the liver and brain. The statistical test performed was a Wilcoxon rank sum test. **C.** Distribution of turnover rates as a function of protein abundance. Protein abundance is binned using groups with an approximately equal number of observations.

bioRxiv preprint doi: https://doi.org/10.1101/2023.05.18.541242; this version posted May 21, 2023. The copyright holder for this preprint (which was not certified by peer review) is the author/funder, who has granted bioRxiv a license to display the preprint in perpetuity. It is made available under aCC-BY 4.0 International license.

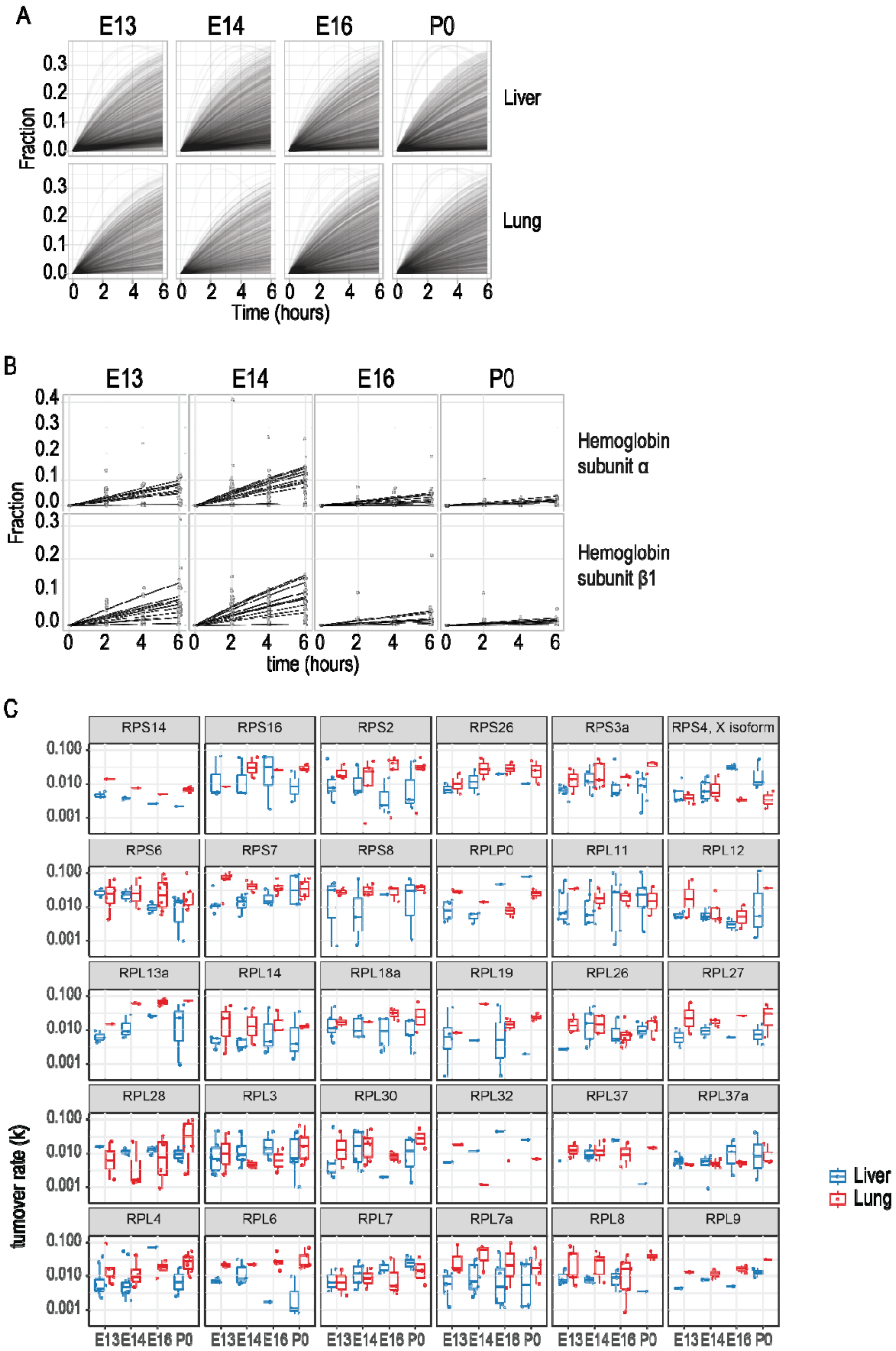

bioRxiv preprint doi: https://doi.org/10.1101/2023.05.18.541242; this version posted May 21, 2023. The copyright holder for this preprint (which was not certified by peer review) is the author/funder, who has granted bioRxiv a license to display the preprint in perpetuity. It is made available under aCC-BY 4.0 International license.

Figure S5: Quantifying turnover rates across mouse fetal development

A. Turnover rate profiles of all peptides for each gestational age and tissue. **B.** Turnover rate profiles showing all the data points and best-fit line for hemoglobin α and β1 in liver tissue. **C.** Summarized turnover rates quantified for ribosomal proteins shared between the liver and lung.

bioRxiv preprint doi: https://doi.org/10.1101/2023.05.18.541242; this version posted May 21, 2023. The copyright holder for this preprint (which was not certified by peer review) is the author/funder, who has granted bioRxiv a license to display the preprint in perpetuity. It is made available under aCC-BY 4.0 International license.

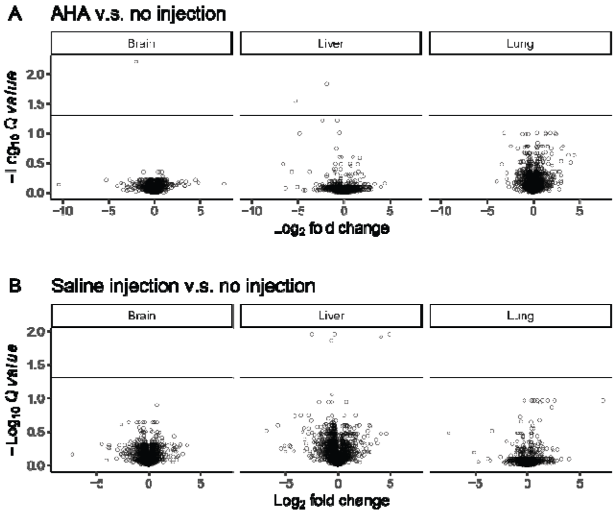

Figure S6: Volcano plots of three organs after AHA and saline injections.

A. Volcano plot comparing the the proteomes after AHA injection and control (no injection). **B.** Volcano plot comparing the the proteomes after saline injection and control (no injection). Statistical analysis was performed using a Student's *t*-test and *p* values were adjusted for multiple hypothesis testing.
